# Clinical and Immunological Analysis of Cutaneous Leishmaniasis before and after Different Treatments

**DOI:** 10.1155/2013/657016

**Published:** 2013-06-13

**Authors:** José A. O'Daly, Humberto M. Spinetti, Joe Gleason, María B. Rodríguez

**Affiliations:** ^1^Astralis LTD, 1076 Stuyvesant Avenue, Irvington, NJ 07111, USA; ^2^Instituto Venezolano de Investigaciones Científicas (IVIC), Altos de Pipe, Km 10 Carretera Panamericana, Caracas 1010A, Venezuela

## Abstract

Amastigotes from *L. (L.)amazonensis* (La), *L. (L.)venezuelensis* (Lv), *L. (V.)brasiliensis* (Lb), and *L. (L.)chagasi* (Lch) were cultured in a free cells liquid culture medium. Patients (*n* = 87) from a cutaneous leishmaniasis (CL) hyperendemic region receiving different treatments were followed up from January 1994 to August 2000. Time for remission of lesions were spontaneous remission (SR) 7 weeks; Glucantime (Glu) chemotherapy 9 weeks; immunotherapy with La, Lv, Lb, and Lch amastigotes Tosyl-Lysil Chloromethyl-ketone (TLCK) treated and Nonidet P-40(NP-40) extracted (VT) 7 weeks. Delayed type hypersensitivity (DTH) response with leishmanine intradermic reaction (IDR) was higher in CL patients than healthy controls (*P* < 0.05) and increased in active secondary versus primary infection (*P* < 0.001) with diagnostic value 1.74 for active infection and 1.81 postclinical remission. Antibodies to amastigotes characterized by Enzyme Linked Immunosorbent Assay (ELISA) decreased in sera postclinical remission versus active infections (*P* < 0.001), with a diagnostic value from 1.50 to 1.84. Immunoblottings antigenic bands frequency as well as Integral Optical Density (IOD) Area Densitometry decreased with sera from SR, after Glu or VT treatments in CL volunteers. Intracellular parasitism is due to normal antibodies recognizing parasite antigens after inoculation by vector. VT vaccine induced mainly cellular immunity, for remission of lesions and protection from CL infection.

## 1. Introduction

Leishmaniasis is a global zoonosis from the tropics and subtropics, with humans serving as accidental hosts. Due to disease prevalence, one-tenth of the world's populations (700 million people) are at risk of infection. Globally, there are 12 million cases, and the incidences of visceral (VL) and CL infections are approximately 0.5 and 1–1.5 million new cases each year, respectively [1, 2]. In a sample population (*n* = 162), parasites were obtained from 85 patients (52.5%), and were isolated from the blood by cultures of 50 patients (30.9%). Isoenzyme analysis confirmed that the organisms in blood and skin were the same, which underlines the invasive potential of the parasite that escaped the skin [3].

Extracellular procyclic promastigotes in the vector mature to metacyclic promastigotes (motile), that evolve to amastigotes (nonmotile) once they enter cells in the vertebrate host after the insect bite. The amastigote eventually evolves back to the promastigote form in the vector after a blood meal in infected hosts, closing the cycle. The mature infective metacyclic promastigotes have surface glycosyl inositol phospholipid (GIPL) and lipophosphoglycan (LPG), virulence factors which inhibit the action of the complement system. Once inside the host, metacyclic promastigotes are taken up by macrophages through binding to complement receptors 1 and 3 or C-reactive protein receptor and, after 24–72 hours, transform into intracellular amastigotes with no surface GIPL or LPG. The amastigotes begin to multiply in the parasitophorous vacuole in the macrophage, suppressing interferon gamma (IFN**γ**) and the production of nitric oxide (NO) and superoxide [4]. 

The immunological response in humans and experimental animals is induced by the amastigote form and not by the promastigote, which enters the host target cell immediately after infection and is not seen by the host immune system. Amastigotes inhibit antigen presentation by repressing the expression of Class I and Class II MHC, both basally and following stimulation with IFN**γ** [5–7]. 

The insoluble antigenic fraction from parasites primarily stimulated CD4+ T cells, while the soluble fraction showed a mixed profile, with CD4+ T cells being the main responsible for Th2 cytokines and CD8+ T cells for Th1 cytokines [8]. 

 Residual parasites remain in the host forever and can be reactivated by AIDS [9, 10]. The challenge is to identify antigens and understand how humoral and cellular immune mechanisms cooperate for immunoprophylaxis, immunotherapy, and clinical remission of lesions [11, 12]. 

The control measures are early case detection and chemotherapy which has been hampered by the toxicity of drugs, severe side effects and by drug resistance in parasites. The development of effective and affordable vaccines against leishmaniasis has not been achieved. Candidate antigens, including killed promastigotes, live attenuated parasites, crude parasites, pure or recombinant leishmania proteins or DNA encoding leishmanial proteins, and immunomodulators from sand fly saliva, have been used; however, very few candidate vaccines have progressed beyond the experimental stage [1, 13].

Increased synthesis of Heat Shock Protein (HSP) occurs in prokaryotic and eukaryotic cells when they are exposed to stress, to protect themselves from lethality, and represent target antigens of the immune response [14]. Interestingly VT vaccine also induced clinical remission of psoriasis [15, 16], psoriatic arthritis [17, 18], and rheumatoid arthritis [19], a serendipity finding [20].

In this paper we present evidence of the immunoprophylactic and immunotherapeutic effects of insoluble proteins from amastigotes, grown in a liquid culture medium without mammalian cells, and the analysis of humoral and cellular immune responses by ELISA and immunoblottings in VT vaccinated and Glucantime treated volunteers before and after clinical remission of lesions in primary and secondary infections in human beings. 

## 2. Materials and Methods

### 2.1. Parasites

The following *Leishmania* strains were used: *L(L)amazonensis* (La: IFLA/BR/67/PH8), *L(L)venezuelensis* (Lv: MHOM/VE/80/H16), *L(V)brasiliensis* (Lb: MHOM/VE/75/H27), and *L(L)chagasi* (Lch: MHOM/BR/74/PP75). Amastigotes were cultured in O'Daly's liquid culture medium without mammalian cells present [21]. First generation polyvalent antigens and second generation monovalent antigens (La, Lv, Lb, and Lch) vaccines were prepared as published [15–20]. The final first generation polyvalent immunogen contained 1 mg/mL or 250 *μ*g of each leishmania specie antigens in PBS supplemented with Rehydragel and 4 *μ*g/mL of gentamicin. The concentration of alumina was 0.25 mL per mg (v/w) of parasitic protein.

### 2.2. Diagnosis of Cutaneous Leishmaniasis

Cases with ulcers or nodules in patients from “El Ingenio” and “La Planta” from January, 1994 to August, 2000 were considered as potential CL patients detected in house per house visits every 15 days. Diagnosis was established by IDR >5 mm with polyvalent leishmanine and ELISA with amastigotes antigens. Concomitant bacterial infections in ulcers were treated with Flucoxaciline (Floxapen) at 100 mg/kg/day orally every 8 hours for 10 days. Patients with IDR >5 mm were selected, a biopsy taken with dermal punch of 6 mm in diameter, from the edge of the ulcer, and aliquots processed for microscopic analysis of amastigotes in macrophages after May-Grunwald-Giemsa staining, injection of 0.1 mL in mice footpads, and parasite culture in O'Daly's medium [21]. Peripheral blood from patients was also taken by venous puncture in the left arm, with Vacutainer for ELISA and immunoblottings. An effective clinical remission was considered the total involution of ulcers or nodules and the appearance of a retractile scar without any inflammatory signs and no relapse for a follow-up period of 6 months after remission.

### 2.3. Treatment of Healthy Volunteers with Amastigotes Proteins from Several Leishmania Species or Glucantime

The study group population (*n* = 87) was in average 25.1 ± 17.1 years old range 5 to 62 years old 45.5% were females and was distributed in several group samples that received different treatments as explained in Tables 1, 2, and 4. Immunoprophylaxis (VT) was applied with amastigotes proteins to susceptible volunteers by personnel from the IVIC immunobiology laboratory, in “El Ingenio” and “La Planta” in Guatire Miranda State, Venezuela, from October to December, 1993. Polyvalent first generation vaccines were injected intramuscularly in the deltoid area in 0.20 mL of PBS containing 200 *μ*g of amastigotes proteins monthly, for three times, one month apart, with previous signature of informed consent. For immunotherapy, volunteers with CL from January, 1994 to August, 2000 with lesions >3 months evolution, received 500 *μ*g/dose of VT, weekly, up to 12 doses intramuscularly in the deltoid region. House per house visit was done every 15 days for finding efficacy of treatment (Tables 1, 2, and 4). Glu treatment was done at the Tropical Medicine Institute, Central University of Caracas (UCV), Venezuela, doses calculated at 10 mg/kg/day and injected during 10 days, with a follow up every 15 days. 

### 2.4. Main Criteria for Trial Inclusion

Eligible subjects included males and females 5 to 62 years of age. For immunoprophylaxis subjects were IDR− and with no previous CL. Additional inclusion criteria were use of a medically accepted form of contraception throughout the study and a negative pregnancy test (females of child-bearing potential). For immunotherapy, IDR+ volunteers with lesions present for at least 3 months were diagnosed and treated as explained above.

### 2.5. Main Criteria for Trial Exclusion

Any female subject pregnant or lactating was excluded from the trial. All trial participants were evaluated for a history of documented immunodeficiency, HIV status, opportunistic infections, or ongoing uncontrolled infections and were a basis for exclusion. Subjects, whose medication involved any vaccines, allergy desensitization products, or use of topical therapy (except emollients) for CL within 2 weeks preceding the first administration of the study medication, were excluded from trial participation. Subjects with a known hypersensitivity to gentamicin or any other ingredient in the study medication, local anesthesia, or diagnostic agents used for tests pertaining to the trial protocol and a documented history of alcohol abuse were excluded from trial participation. 

### 2.6. Delayed Type Hypersensitivity

The IDR skin test with leishmania antigens provides a DTH reaction and is a good marker of the cellular immune response in leishmaniasis. Leishmania skin test solutions (polyvalent leishmanine) were prepared with equal concentrations of four Leishmania species (La, Lv, Lb, and Lch). Also, monovalent leishmanine was prepared for each *Leishmania* species. The final concentration of the solution in each case was 40 *μ*g/mL in PBS. Four *μ*g in 0.1 mL was injected intradermally in the volar surface of the right arm. The reaction was measured 48 hours later by the ball-point-pen technique [22]. A diameter >5 mm was considered a positive IDR.

### 2.7. SDS-Polyacrylamide Gel Electrophoresis

50 *μ*g of each leishmania specie for Coomassie blue staining and 60 *μ*g for silver staining were electrophoresed in invitrogen 4–20% NuPAGE Tris-acetate SDS-PAGE precast gels under reducing conditions with Tris-glycine SDS running buffer in a XCell SureLock Mini-Cell (Invitrogen life technologies) following the manufacturer's instructions as published [16, 19, 23]. Proteins were stained with SilverXpress silver or Coomassie brilliant blue (R-250) staining kits (Invitrogen) and analyzed in a BIORAD image system with Quantity One 1D analysis Software. Prestained molecular weight markers (Biorad) were used in each gel.

### 2.8. ELISA

Antileishmania antibodies to amastigotes in sera from volunteers were performed before and after treatments, as published [17, 19, 20, 23]. The antigens were derived from amastigotes of the four leishmania species that constitute VT drug substance, tested as polyvalent extracts, and separately not pooled, as found in the VT drug product (La, Lv, Lb, and Lch). The wells of each ELISA plate were coated with a solution containing 200 *μ*g of antigens per mL. After drying, plates were washed with PBS containing 0.05% Tween 20 and postcoated with PBS containing 0.05% Tween 20 and 1% bovine serum albumin. The plates were washed, and serum samples (diluted 1 : 1,000) in PBS were added to each well and incubated. Another wash was followed by the addition of antihuman IgG horseradish peroxidase linked (Fab′)_2_ fragment from sheep and incubation overnight. A final wash was followed by the addition of the substrate ABTS (2-2′-azino-di-benzothiazoline sulfonate) and 10 *μ*L H_2_O_2_. The assay was read in an automatic ELISA reader (Titertek Multiskan Plus) at 450 nm. A bovine serum albumin calibrator diluted from 6.25 to 400 ng/mL was added to assigned wells. Any replicate wells with an average of 3 standard deviations above the average concentration recorded for the negative controls (100 ng/mL), was considered a positive reaction. All values represent the average of duplicate assays of three different experiments performed with sera of patients and healthy controls IDR−. Protein concentration was determined by Lowry or BCA [24, 25].

### 2.9. Immunoblottings

50 *μ*g in 10 *μ*L of each leishmania specie was electrophoresed in invitrogen 4–20% NuPAGE Tris-acetate SDS-PAGE precast gels under reducing conditions with Tris-glycine SDS running buffer in a XCell SureLock Mini-Cell (Invitrogen life technologies) as published [17, 19, 20, 23]. Afterwards transfer to nitrocellulose paper (Schleicher & Schull, Keene, NH, USA) was performed in a Mini-trans-Blot (Biorad) following manufacturer's instructions. After reaction with the primary antibody from various patients' sera and controls, staining was done with a secondary anti-human immunoglobulin horseradish peroxidase linked (Fab′)_2_ fragment from sheep, (Amersham, UK) at 1 : 1000 dilution for 2 hours at room temperature. Finally after a wash with SST (Tris-HCl 0.05 M, 0.15 M NaCl, pH 9.5)-tween 20, a solution with 0.05% diaminobenzidine (w/v), 0.03% H_2_O_2_ (v/v), and 0.03% CoCl was added, waiting until color was developed, washed with distilled water, and photographs were taken [23]. Protein concentration was determined by Lowry or BCA [24, 25]. Immunoblottings after staining were aligned side by side from each patient's sera, digitalized with a scanner scan jet 4C and Deskcan II, 2.3 program (Hewlett Packard 1991–1995) and then analyzed with Gel-pro 98, 3.0 Media Cybernetic 1995-1996. The integrated optical density (IOD) values corresponding to the area under the curve from different bands was obtained and coefficient of variation calculated before and after clinical remission as follows: OD posttreatment/OD pretreatment. Values >1.0 revealed increase in OD antigens and values <1.0 decrease in OD antigens after treatment. OD was proportional to IgG primary antibody from sera bound specifically by each leishmania spp. antigens between 13 and 150 kDa and revealed by sera from patients and controls with secondary mouse anti-IgG (Fab′)_2_ antibody.

### 2.10. Statistical Methods

Treatment groups were compared using an analysis of variance (ANOVA) model with treatment as a fixed effect. To control for multiple comparisons, the hypothesis of an overall treatment effect was tested first. If the treatment effect *P* value was <0.05, then pairwise comparisons of each active treatment group versus control were performed using contrasts. The ANOVA assumption of normality and homogeneity of variances was tested, and, where appropriate, a nonparametric approach (Wilcoxon test, Tukey's multiple comparison tests) was used to compare treatment groups. Student' *t*-test was also used. All calculations were done with GraphPad Prism software.

## 3. Results

### 3.1. Patient Demographics

The study group sample (*n* = 87) from “La Planta” a hyperendemic region for CL, prevalence 24.8 o/oo, had lesions in uncovered areas from the body remitting with different treatments in six years of followup. Similar percentages of patients had scars (49.25%) and ulcers (49.25%) after primary infection, while 1.5% had skin nodules. Unique ulcers were found in 82.57% while multiple ulcers in 17.43% patients. The ulcer's area had average 8.62 ± 7.65 cm^2^ with a range from 0.44 to 33.18 cm^2^. 170 primary lesions were distributed in the study group as in Table 1; their evolution had average 2.37 ± 3.32 months, range from 15 days to 9 months. 

### 3.2. Chemotherapy and Amastigotes Vaccine Treatments in Patients with CL after Primary and Secondary Infections

Treatments were distributed in 87 patients with primary lesions as follows: 35% had SR of lesions, 42% received Glu, and 21% VT vaccine. Seven weeks was chosen as golden standard for remission time, selected from patients with natural SR of CL without any treatment. Patients treated with Glu had 9 weeks and with VT 7 weeks for remission, similar to the golden standard (Table 1). Two patients without remission post-Glu received 7 ± 1 VT doses and remitted in 7 weeks. Patients who received VT had 6 doses for total remission in 7 weeks, without relapses in 6 years of followup (Table 1). 

Patients with secondary infections (*n* = 32) had mean age 24.69 ± 19.06 years old, range 5 to 62 years old; 46.87% were females, and scars were found in all patients. Time between remission and relapses had average 30.14 ± 27.43 months and range 1–111 months. In four patients relapses appeared at the edge of the scars induced by the primary infection. Forty scars had total area 7.94 ± 7.38 cm^2^, range 1.9–30.0 cm^2^, unique scars 65%, and multiple scars 35%, mainly in the right leg. Together with scars, 37 new lesions appeared at relapses 87.5% were ulcers and 12.5% nodules. Unique ulcers (75%) predominated over multiple ulcers (12.5%) and unique nodules (9.4%), over multiple nodules (3.1%) with similar percentages in arms and legs (Table 2). In secondary infections, four patients with new lesions had SR time in 8 weeks, and 7 patients treated with Glu had also remission in 8 weeks (Table 2). The four patients with SR had relapses, three had SR again in 4 weeks, one needed 10 Glu doses and remitted in 10 weeks. In the group of 7 patients treated with Glu in secondary infections, 4 had relapses that remitted without treatment in 6 weeks, and 3 needed 25 new doses of Glu remitting in 6 weeks (Table 3).

### 3.3. Delayed Type Hypersensitivity with Polyvalent Leishmanine after CL Primary and Secondary Infections

Cellular immunity was analyzed by DTH response in skin, comparing the IDR, between active and SR of lesions. The cutoff point to consider a reaction as positive was diameter >5 mm. IDR was higher in active secondary infections and significantly different to patients with active primary infections (*P* < 0.001), while in patients of post-clinical remission values were similar (*P* = 0.96). In 127 healthy volunteers without previous CL we found 22.04% IDR positivity in the study group (Table 4).

The vaccinated volunteers IDR− exhibited higher IDR post-VT (*P* < 0.001) while IDR was similar in IDR+ volunteers (*P* = 1.0). Nonvaccinated controls had higher IDR in IDR+ volunteers similar to data above (Table 5).

The diagnostic value (specificity + sensitivity) was 1.76 for patients with active infection and 1.81 post-clinical remissions, which underlines the fact of DTH as positive tool for diagnosis of CL with polyvalent amastigotes antigens leishmanine (La, Lv, Lb, and Lch) after TLCK treatment and NP-40 extraction.

### 3.4. Incidence of CL according to IDR Status in VT Vaccinated Volunteers and Nonvaccinated Controls

Majority of CL cases (59%) with parasitological diagnosis were in sixty-nine IDR− nonvaccinated volunteers. In this group 26% had SR, and 73% received Glu. In IDR+, nonvaccinated volunteers five CL cases (27%) were found, four remitted spontaneously and only one received Glu for clinical remission. Nine CL cases (14%) were found in sixty-one IDR− post-VT vaccinated volunteers, 4 patients had SR and 5 received Glu treatment. In ten IDR+ post-VT vaccinated volunteers three CL cases were found, one received Glu treatment only, and the other two remitted spontaneously. All patients were observed in six years of followup (Table 6). 

### 3.5. Humoral Immunity in CL after Primary and Secondary Infections with Monovalent Amastigotes Antigens

**Figure 1:**
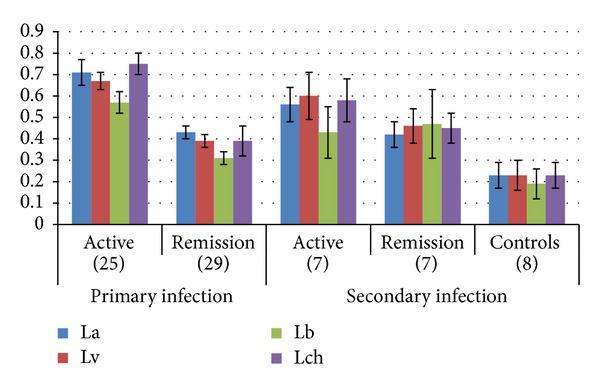
ELISA with monovalent amastigotes antigens, sera from patients with active cutaneous leishmaniasis and postspontaneous remission of lesions in primary or secondary infections as compared to sera from controls (patients). La: *L(L)amazonensis*; Lv: *L(L)venezuelensis*; Lb: *L(V)brasiliensis*; Lch: *L(L)chagasi. *

The cutoff point to consider a value as positive was established as the average OD in IDR(−) controls+3 standard deviations as follows: La = 0.41, Lb = 0.40, Lv = 0.44, and Lch = 0.41. In primary infections, humoral response was higher with sera from patients with active CL than after SR (*P* < 0.001) of lesions, and the latter values are all equal or below the cutoff point. In secondary infections values were similar (*P* > 0.05). The diagnostic value in active infection was La = 1.81, Lv = 1.84, Lb = 1.50, and Lch = 1.84, evidence that monovalent antigens could be used in ELISA as a diagnostic tool for CL (Figure 1). 

**Figure 2:**
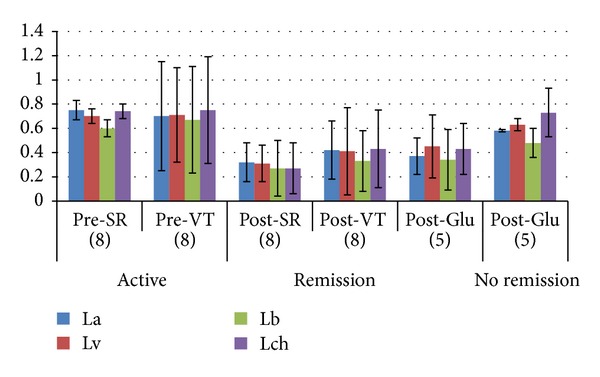
ELISA obtained with monovalent amastigotes and sera from patients with primary infection, with active lesions or remission after SR, VT or Glu treatments. (Patients).La:* L(L)amazonensis*; Lv: *L(L)venezuelensis,*; Lb:* L(V)brasiliensis*; Lch:* L(L)chagasi. *No significant correlation was found between IDR and ELISA values in 32 patients with CL. The correlation indexes were for La: 0.0098 (*P* = 0.588); Lv: 0.0057 (*P* = 0.88); Lb: 0.012 (*P* = 0.535); Lch: 0.026 (*P* = 0.372). OD values were: Pre-SR: before spontaneous remission. Pre-VT: before proteins from Leishmania amastigotes after TLCK and NP-40 treatment. Post-SR: after spontaneous remission. Post-VT: after proteins from Leishmania amastigotes after TLCK and NP-40 treatment. Post-Glu: after Meglumine antimoniate or Glucantime® treatment.

La:* L(L)amazonensis*; Lv: *L(L)venezuelensis*; Lb:* L(V)brasiliensis*; Lch:* L(L)chagasi. *OD values between active pre-SR and pre-VT were not statistically significant (*P* > 0.05) and were higher than values post-clinical remission of lesions. Similar values were obtained in patients with remission in post-SR, post-VT and post-Glu treatment (*P* > 0.05), which confirmed that treatments with VT or Glu were effective and similar to natural SR (golden standard) in CL patients. Interestingly, OD values post-Glu in patients that had no remission of lesions were higher than post-SR, post-VT or post-Glu with remission of lesions (*P* < 0.05), pointing out ELISA as a good tool to follow up evolution of CL infection (Figure 2).

No significant correlation was found between IDR and ELISA values in 32 patients with CL. The correlation indexes were for La: 0.0098 (*P* = 0.588); Lv: 0.0057 (*P* = 0.88); Lb: 0.012 (*P* = 0.535); Lch: 0.026 (*P* = 0.372). 

### 3.6. Humoral Immunity with Amastigotes Antigens in CL and Other Tropical Diseases

**Figure 3:**
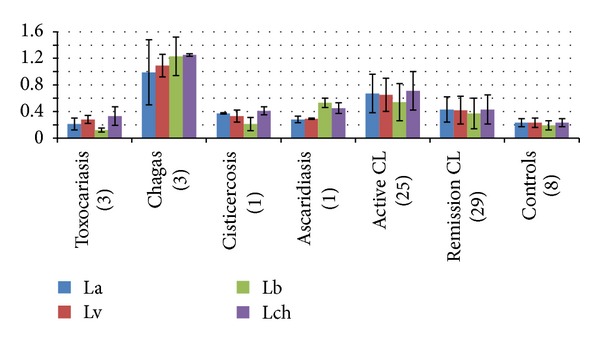
ELISA in patients with cutaneous leishmaniasis and other tropical diseases with complete amastigotes antigens^a^ (patients). La: *L(L)amazonensis*; Lv: *L(L)venezuelensis*; Lb: *L(V)brasiliensis*; Lch: *L(L)chagasi. *

ELISA with sera from patients with other tropical diseases showed that in Toxocariasis, Cisticercosis, and Ascaridiasis OD values versus controls were not significant (*P* > 0.05). On the contrary, sera from Chagas disease and/or active CL had higher significant (*P* < 0.05) values versus controls with all leishmania amastigotes antigens. OD values post-clinical remission of CL were lower than active CL, close to the cutoff value, similar to data above (Figure 3).

### 3.7. Immunoblottings with Amastigotes Antigens and Sera from CL and Controls before and after Different Treatments

Another tool to define important antigens in CL is the analysis of bands' frequency in immunoblottings of several leishmania species, with sera from active infection and after clinical remission of lesions. Antigens 13 to 145 kDa revealed by 80–100% sera were selected as the most immunogenic proteins. A band was considered present if it had an optical density >10 units by densitometry analysis. 

Leishmania species in the study group had a characteristic set of antigens. Antigenic bands revealed by sera from patients with active CL and post-clinical remission in the same patients were compared within the same group with IDR(−) controls (i.e., La in active or remission versus La in IDR− controls, Lb in active or remission versus Lb in IDR− controls, and so on with Lv and Lch). Common bands present in active, remission, and control IDR− groups are in red bold numbers. MW bands revealed by sera from active and clinical remission patients, not found by sera from controls, at the respective homologous leishmania specie are in bold blue numbers (Table 5). This is evidence that amastigote antigens sensitized B cells receptors and expanded specific parasites' clones in active and after clinical remission of lesions not revealed by healthy control sera. Total number of antigens decreased postclinical remission in all species as compared to patients with active lesions (Table 5). The shared common antigens by controls and patients explain the location of intracellular amastigotes in the vertebrate host, since promastigotes are immediately recognized by normal immunoglobulin after injection of parasites by the vector in the skin and afterwards phagocytose by APC, neutrophils, and macrophages. Specific amastigotes antigens not found by control sera and recognized by sera from active lesions were as follows: 8 in La; 11 in Lb; 4 in Lv; 6 in Lch. In bold green numbers specific antigens found in one leishmania specie only, had the following MW in active lesions: La: 132; Lb: 115 kDa; Lv: none; and Lch: 128 kDa; after clinical remission in the group MW were: La: 100; Lb: 108; Lv: 80; and Lch: 86 kDa, respectively. In bold black numbers antigens MW 45 kDa were found in controls in La, Lb, Lv, and Lch by 100% sera, also MW 96 kDa in Lb by 100% control sera. Both antigens were not recognized by any patient's sera (Table 7), only by healthy controls. In normal back numbers, MW 86 kDa in La and Lv, 90 kDa in La and 128, and 132 kDa in Lb are in 80–100% controls sera but not in sera from CL patients in the respective leishmania group.

Bands frequency in the SR natural process revealed by sera from four volunteers showed fourteen antigens (56%) MW 142, 132, 128, 120, 115, 104, 100, 98, 90, 86, 80 53, 45, and 27 kDa decreasing after remission of lesions. Only one antigen (4%) MW 33 kDa increased in band frequency; the rest ten antigens (40%) MW 136, 108, 74, 65, 60, 57, 50, 40, 35, and 22 kDa frequency remained unchanged. Band frequency before and after Glu revealed by sera from three volunteers, showed twelve antigens (41.4%) 145, 132, 128, 104, 100, 70, 53, 33, 31, 24, 17, and 15 kDa decreasing while four antigens (13.8%) 83, 80, 57, and 29 kDa increased after remission of lesions, the rest thirteen antigens (44.8%) 98, 86, 78, 74, 65, 60, 50, 43, 40, 35, 27, 22, and 20 kDa remained unchanged. Band frequency before and after VT revealed by sera from 3 volunteers showed twelve antigens (37.5%) 142, 120, 96, 80, 57, 35, 29, 22, 20, 17, 15, and 13 kDa decreasing while two antigens (6.25%) 90 and 60 kDa increased after remission of lesions; the rest of eighteen antigens (56.25%) 145, 136, 132, 128, 122, 115, 104, 100, 98, 86, 74, 65, 50, 45, 40, 33, 27, and 24 remained unchanged. Decrease in bands' frequency predominated in low MW antigens from 57 to 13 kDa in post-Glu and post-VT treatment in comparison to natural SR. Molecular weights bands 80 to 145 MW also decreased, eleven antigens post-SR, followed by six post-Glu and four post-VT (Table 8).

### 3.8. Densitometry in Immunoblottings of *L(L)amazonensis* Amastigotes before and after Remission of Lesions

Coefficient of variation was calculated as

(1) $$\begin{array}{c} \frac{\text{Area}\;\;\text{curve}\;\;\text{OD}\;\;\text{Post-treatment}}{\text{Area}\;\;\text{curve}\;\;\text{OD}\;\;\text{Pre-treatment}}. \end{array}$$

Integrated Optical Density (IOD) area values >1.0 correspond to increase in antigen density, while values < 1.0 corresponds to a decrease in density, respectively. Optical density was proportional to IgG primary antibody from sera pre- and posttreatment, bound by each leishmania specie amastigotes antigens between 13 and 145 kDa, and revealed with secondary mouse antihuman IgG (Fab′)_2_ antibody. 

**Figure 4:**
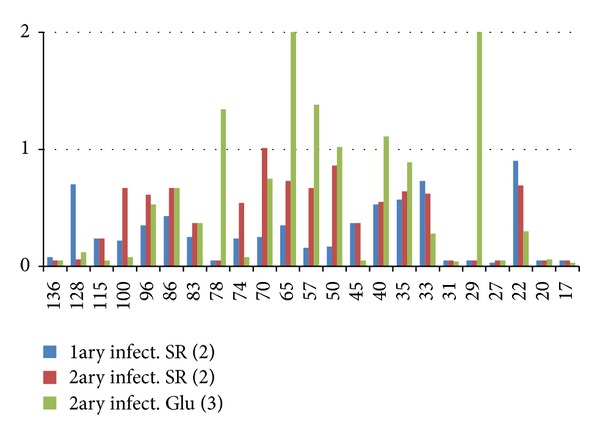
IOD area values in spontaneous remission (SR) after primary infection (infect.) SR and post-Glu treatment after secondary infection (patients). Standard deviations were 5–20% of average and are not shown for the sake of clarity.

The IOD from amastigote antigens revealed by sera from SR decreased significantly in quantitative immunoblottings in primary (100% antigens) and secondary (94% antigens) infections, after clinical remission of lesions, due to low antibody concentration once the active infection was over and parasites under control. Sera from SR had only one antigen MW 70 kDa with IOD increasing postclinical remission in secondary infections. Sera post-Glu in secondary infections had increased IOD area in six antigens (26.1%) MW: 78, 65, 57, 50, 40, and 29, and the rest seventeen antigens (73.9%) decreased in area density (Figure 4).

**Figure 5:**
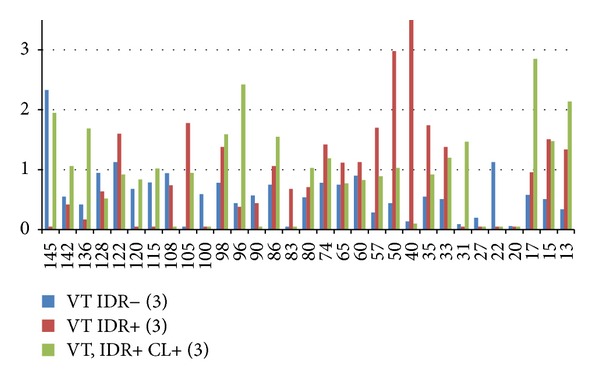
IOD area values in VT vaccinated volunteers after primary infection in intradermic reaction (IDR) negative, IDR positive, and IDR+ with diagnosis of cutaneous leishmaniasis (CL) (patients). Standard deviations were 5–20% of average and are not shown for the sake of clarity

 After primary infection in VT vaccinated volunteers, (1) sera from previous IDR− volunteers had decreased in IOD area in twenty-eight antigens (90.3%), and three antigens (9.7%) MW 145, 122, and 22 kDa increased only. (2) Sera from previous IDR+ volunteers with subclinical infections had seventeen antigens (54.8%) which decreased IOD values while in the rest fourteen antigens (45.2%), valuesin IOD area increased. (3) Sera IDR+ with previous healed CL had decrease in IOD in sixteen antigens (51.6%) while the rest fifteen antigens (48.4%) had increase in IOD (Figure 5).

## 4. Discussion

### 4.1. Amastigotes Treated with TLCK Inducing CL Clinical Remission

Hamsters immunized with TLCK treated Lb amastigotes from culture, infected with hamsters Lb amastigotes presented (1) a gradual increase in T and B cell responsiveness to mitogens by lymph node lymphocytes, with an increased response to concanavalin A (ConA); (2) no changes in response to dextran sulphate and pokeweed mitogen in splenocytes; (3) absence of parasites in lymph nodes after 6-week postinfection and a nodule 4 times smaller than that of infected control animals, which was undetectable 70 days after infection. Hamsters preimmunized with TLCK treated Ld amastigotes from culture did not show suppression of the blastogenic response to mitogens of spleen and lymph node cells after infection with Ld amastigotes from hamster's spleen and survived for more than one year, whereas infected, unimmunized animals died five months after infection. Animals preimmunized with culture parasites (Lb or Ld) treated with phenylmethyl sulphonyl fluoride (PMSF) and infected with Lb or Ld amastigotes did not show any protective effect [26]. 

### 4.2. Intradermic Reaction as Useful Tool for CL Diagnosis

The IDR with polyvalent VT had a diagnostic value >1.0 for CL surveillance useful to know the prevalence and incidence in endemic and hyperendemic regions. Positive IDR in residents living in endemic areas without previous CL in skin are defined as subclinic infections. In Corguinho, Mato Grosso do Sul, Brazil, an endemic region for Lb, 15.7% had positive IDR to leishmanine with no previous infections [27]; also in endemic regions for *L(L)peruviana* in Lima, Ancash, and Piura in Peru, 17.0% IDR+ cases were recorded [28] and in Northeast Ecuador with high prevalence of *L(L)guyanensis* and *L(L)panamensis* 17.2% positivity was observed [29]; all values are in agreement with data in Venezuela: 22.0% IDR+ volunteers to polyvalent amastigote leishmanine in healthy subjects, with no previous CL infection. The association of protective immunity with IDR response and cellular immunity explains the higher incidence of CL in IDR− (59%) as compared with IDR+ (27%) volunteers in the study group. 

### 4.3. Parasite Proteolytic Enzymes in Leishmania Virulence

Proteases play essential roles in the pathogenesis of leishmaniasis, helping parasites to invade and multiply in mammalian host cells. However, few studies have addressed serine proteases in leishmania infection and their role in host pathogenesis. The level of internalization of parasites into host macrophages after treatment amastigotes with the anti-115 kDa antibody was significantly reduced, suggesting that this serine protease probably plays a role in the infection process [30]. The basis of VT vaccine is inhibition of serine proteases with TLCK, which induced immune prophylaxis and immunotherapy in CL patients. Interestingly other protease inhibitors as PMSF had no effect [26].

Prohibitin is concentrated at the surface of the flagellar and the aflagellar pole, through which host-parasite interactions occur. Overexpression of wild-type prohibitin increases infectivity in parasites [31]. VT vaccine does not have prohibitin since amastigotes were extracted with NP-40 and supernatant discarded; thus no antibodies or cellular immunity to surface proteins could be found [17–20].

### 4.4. Decrease in Humoral Immunity Inducing CL Clinical Remission

Specific antibodies decreased post-clinical remission of lesions, confirmed by ELISA, bands' frequency, and IOD bands' area in immunoblottings, pointing out to the fundamental role of cellular immunity in controlling infection. Three months was the time waited in CL patients to start VT or Glu treatments to avoid the self-healing leishmaniasis effect. Size of skin ulcers in relapses was lower than in primary lesions; thus, a vaccine was possible to control CL. VT vaccine had remission time evidence VT amastigotes vaccine was useful for CL treatment. Furthermore VT vaccinated subjects had lower CL cases in IDR− and IDR+ volunteers than IDR− nonvaccinated subjects. Specific IgG antibody titer decreases SR in comparison to active CL [32]. Antibodies to *L(L)infantum* might persist for many years and decrease slowly. The persistence of these specific antibodies and an acute increase in their levels might be a sentinel of a VL relapse [33]. ELISA has been used for diagnosis of CL using soluble (SF) and membrane enriched (MF) antigen fractions obtained from Lb. Sensitivity was 89.5% for each fraction, and specificity was 89.5% for SF and 93.4% for MF [34]. Similar results were obtained with VT vaccine insoluble amastigote antigens from four species Lb, Ld, Lv, and Lch. ELISA with monovalent amastigotes evidenced that antibodies were induced on B cells in primary active infection and decreased significantly post-clinical remission, once parasites were under control. This underlines that cellular immunity played a key role in control of parasites in secondary infection as evidenced by a high positive DTH in active and postclinical remission of lesions. ELISA in active lesions, pre-SR and pre-VT was similar in primary infection evidencing that B cell clones were activated and reacting to all monovalent amastigotes. Antibodies decreased significantly post-SR, post-VT, and post-Glu treatment, since VT was stimulating mainly cellular immunity. Interestingly antibodies did not decrease in one group of patients without clinical remission post-Glu treatment. The OD values in this group, compared with values that had clinical remission, were significant (*P* < 0.05), a very good internal control. The reason why these patients could not switch on cellular immunity and decrease humoral immunity is under investigation.

Microorganisms replicate in macrophages, facilitated by surface IgG antibody increasing intracellular infection through Fc*γ*-receptor by phagocytosis. The ligation of monocyte or macrophage Fc*γ* receptors by IgG immune complexes, suppresses innate immunity, increases production of IL-10, and bias T-helper-1 (Th1) responses to Th2 responses, leading to increased infectious output by infected cells [35, 36]. Normal IgG antibodies recognized many parasite antigens, isolating them from the immune system, directing promastigotes to the intracellular site. Cellular immunity in chronic infection was probably mediated by a CD8+ T cells and Th1 response once intracellular amastigotes were under control and lesions disappeared. Parasites remain in the host forever, in proliferative equilibrium inside cells, without cells' lysis, controlled by a permanent cellular immunological response, which once it is stopped, parasites can be reactivated and disseminated as in AIDS [9, 10].

Bands frequency analysis revealed decreased numbers of bands in 56% antigens recognized by sera after SR, 41% post-Glu, 37% post-VT, respectively, explaining OD decreasing in ELISA, in SR and after Glu or VT treatments. The highest decrease was in low molecular weight antigens post-Glu and Post-VT treatment. Decrease in bands' frequency and serum antibodies may be explained by inhibition of antigen presentation by repression of Class I and Class II MHC on infected host cells [5, 6], by interfering with the loading of antigens onto the MHC class II molecule [37] or sequestering the MHC II molecules and antigens within the phagolysosome [38].

The IOD area in amastigote antigens revealed by sera from SR volunteers and post-Glu treatment decreased significantly, and only one antigen 70 kDa increased after SR in secondary infections. Sera post-Glu had increased IOD area in low MW antigens 78, 65, 57, 50, 40, and 29 kDa (26%) after clinical remission of lesions. This was probably due to parasites' death and liberation of low MW antigens to the host, inducing B cell sensitization, antibody production, and immune protection, similar to findings in immunized and infected BALB/c mice [39]. After VT vaccination, IOD area in majority of proteins decreased in IDR− patients, while low and high MW antigens increased in IDR+ subclinical infections, and in IDR+CL+, VT vaccinated volunteers with previous healed CL suggesting VT vaccination also killed parasites inducing NK cells liberating antigens in subclinical and clinical infections, similar to Glu treatment. 

### 4.5. Intracellular Parasitism in Leishmania Parasites

To recognize amastigote antigens, no previous lymphocyte sensitization is required. Leishmania promastigotes sonicates induced *in vitro* proliferation and IFN*γ* production in peripheral blood mononuclear cells (PBMC) from individuals that never had contact with leishmania parasites. The proliferating T cell population was CD2+ in a frequency <1 : 10,000 a response that could be abolished after depletion of CD45RO+ memory cells from the PBMC [40]. Thus, normal CD2+ T cells and normal immunoglobulin from healthy volunteers that never experienced leishmania infection reacted with leishmania antigens in the immunoblotting assay. Interestingly psoriatic patients, DTH negatives to amastigote antigens were also recognized in the blastogenic assay leishmania antigens and were distributed in two groups, low and high responders to amastigote antigens before treatment [16]. Furthermore, a high DTH response with Lb and Lch protein chromatography fractions *in vivo* after treatment with VT polyvalent vaccine was found in human volunteers, confirming the absence of immunosuppression. The lymphocyte stimulation indexes obtained by protein fractions were 50–70% lower than the values induced by intact living leishmania amastigotes [16]. This suggests *in vitro* stimulation appears to be selectively focused on a particular subset of lymphocytes which may be regulatory CD8+ T cells [17–20, 41].

### 4.6. Leishmania Amastigotes TLCK Treated Inducing Clinical Remission of Other Diseases

Surprisingly, VT amastigotes antigens induced clinical remission of all forms of psoriasis [16], psoriatic arthritis (PsA) [17], and rheumatoid arthritis [20] in human volunteers and collagen induced arthritis in mice [19] a serendipity finding [20]. The effect of VT was analyzed in patients with PsA after treatment, finding that C-reactive protein (CRP), complement 5a, TNF*α*, and 1L-1 *β* decreased in human volunteers [20]. Interestingly, proliferation of cutaneous T cell lymphoma *in vitro* was also inhibited by VT monovalent Lch amastigotes antigens in a dose/response relationship [20], which open new roads for the application of this VT treatment to wide spectra of diseases.

### 4.7. Production Costs VT Vaccine

Due to genetic diversity in target populations, including both dogs and humans, a multiple-antigen vaccine will likely be essential. However, the cost of a vaccine to be used in developing countries must be considered. The polyprotein (KSAC) was found to be immunogenic and capable of inducing protection against *L(L)infantum*, responsible for human and canine VL, and against *L(L)major*, responsible for CL. It is a cost-effective vaccine capable of protecting both humans and dogs against multiple *Leishmania* species [42]. The VT polyvalent vaccine for CL, ML, and VL has a very low cost of production, is feasible to be applied to developing countries worldwide.

### 4.8. Heat Shock Proteins and Mechanism of Action of TLCK Treated Amastigotes

HSP long-term confrontation of the immune system similar in the host and microbes invaders may convert the immune response against these host antigens and promote and/or decrease autoimmune diseases including psoriasis [43–45]. There is evidence that recognition of self-HSP60 can have beneficial effects in arthritis and may offer new strategies for improved control measures in the inflammatory processes by administration of peptides cross-reactive to self-determinants [14]. HSP60, HSP70, and Gp96 function as host derived ligands for toll-like receptors (TLR2) and play a role in the pathogenesis of RA and psoriasis [44, 45]. Leishmania antigens are produced after a heat shock in promastigotes that become amastigotes in a liquid culture medium [21]. The MW of chromatography fractions from Lb that induced remission of psoriasis was similar to the range of most HSP host ligands (50–70 kDa) and could be inhibiting the symptoms of psoriasis [15], PsA [17], and CIA [19, 20] by competing with peptides in the respective receptors. Similar mechanisms could be acting in immunoprophylaxis and immunotherapy of CL as shown in this paper. 

## 5. Conclusions

Amastigotes from four leishmania species, cultured in a free cells culture medium without cells treated with TLCK (VT) induced clinical remission of CL after six 500 *μ*g/dose, weekly, up to 12 doses intramuscularly in the deltoid region in 7 weeks and protected volunteers from CL in six years of followup. Humoral immunity decreased after clinical remission of lesions, measured by antibodies in ELISA, frequency of antigenic bands, and density area densitometry in immunoblottings. Intracellular parasitism is due to normal antibodies recognizing parasite antigens postinoculation by vector. VT vaccine induced mainly cellular immunity, measured by DTH intradermic reaction, postremission of lesions as compared to active infection. No patients had relapses or secondary infections after VT treatment in primary infections. VT vaccine induced clinical remission of all forms of psoriasis, PsA, and rheumatoid arthritis in human volunteers and collagen induced arthritis in mice, a serendipity finding, and inhibited proliferation of cutaneous T cell lymphoma *in vitro* which open new roads for the application of this vaccine to wide spectra of diseases.

## Figures and Tables

**Table:** (a)

Localization of 170 lesions, *n* = lesions, *n*/170 = (%)						
Right leg	Left leg	Right arm	Left arm	Abdomen	Thorax	Head
69 (40.58)	43 (25.29)	22 (12.94)	15 (8.82)	6 (3.52)	7 (4.11)	8 (4.70)

**Table:** (b)

Treatments in 87 patients with primary lesions, *n* = patients, *n*/87 = (%)			
Treatments	Doses	Remission	Remission weeks
SR^a^ 31, (35.63)	None	(100%)	7.41 ± 2.71
Glu^b^ 37, (42.52)	24.54 ± 15.07	35/37, (94.59%)	9.02 ± 5.04
VT^c^ 19, (21.83)	6.36 ± 3.41	(100%)	7.40 ± 2.70

^a^SR: spontaneous remission. ^b^Glu: meglumine antimoniate or Glucantime.

^c^VT: *Leishmania* amastigotes (La, Lv, Lb, and Lch) after TLCK treatment and NP-40 extraction.

**Table 2:** Lesions in patients (*n* = 32) with cutaneous leishmaniasis secondary infections.

Localization of 40 scars after primary infection, *n* = scars, *n*/40 = (%)						
Right leg	Left leg	Right arm	Left arm	Abdomen	Thorax	Head
14 (35)	10 (25)	3 (7.5)	5 (12.5)	2 (5)	2 (5)	4 (10)

Localization of 37 new lesions *n* = lesions, *n*/37 = (%)						
Right leg	Left leg	Right arm	Left arm	Abdomen	Thorax	

11 (29.72)	8 (21.62)	7 (18.91)	8 (21.62)	None	3 (8.1)	

**Table 3:** Evolution of lesions in patients (*n* = 32) with cutaneous leishmaniasis secondary infections.

Evolution of lesions after treatments, *n* = patients, *n*/32 = (%)					
New lesion			Relapses		
Patients	Doses	Remission, weeks	Patients	Doses	Remission, weeks
SR^a^ 4 (12.5)	None	8.50 ± 3.41	3 (9.37)	None	4.7 ± 3.0
			1 (3.12)	10, Glu^b^	10
Glu^b^ 7 (21.87)	27.4 ± 16.1	8.71 ± 2.49	4 (12.5)	None	6.33 ± 1.52
			3 (9.37)	25 ± 10, Glu^b^	6.5 ± 3.79

^a^SR: spontaneous remission. ^b^Glu: meglumine antimoniate or Glucantime.

**Table 4:** Intradermic reaction with polyvalent amastigotes leishmanine, in patients with cutaneous leishmaniasis after spontaneous remission of lesions.

IDR^a^ in mm average ± standard deviation (patients)					
Active		Postclinical remission		Healthy controls	
Primary infection (45)	Secondary infection (20)	Primary infection (60)	Secondary infection (13)	IDR− (99)	IDR+ (28)
9.70 ± 0.66	15.60 ± 0.72	14.68 ± 0.99	14.76 ± 0.06	0.12 ± 0.69	10.4 ± 4.98

^a^The cutoff point to consider an IDR positive was diameter >5 mm.

**Table 5:** Delayed type hypersensitivity in intradermic reaction positive and negative volunteers in VT vaccinated and controls.

IDR^a^ in mm polyvalent leishmanine average ± standard deviation (patients)					
Nonvaccinated controls		VT^b^ vaccinated volunteers			
IDR− (69)	IDR+ (18)	IDR− (61)		IDR+ (10)	
		Pre-VT	Post-VT	Pre-VT	Post-VT
0.07 ± 0.58	10.41 ± 5.35	0.18 ± 0.8	9.03 ± 3.33	10.38 ± 4.53	13.95 ± 6.42

^a^The cutoff point to consider an IDR positive was diameter >5 mm.

^b^VT: Leishmania amastigotes (La, Lv, Lb, and Lch) after TLCK treatment and NP-40 extraction.

**Table 6:** Incidence of cutaneous leishmaniasis in IDR− and IDR+ nonvaccinated and vaccinated volunteers.

Cases of localized cutaneous leishmaniasis and treatment; *n* = patients, (%)				
	Nonvaccinated controls		Vaccinated volunteers	
	IDR−	IDR+	Post-VT^c^	Post-VT
	41/69 (59.42)	5/18 (27.7)	9/61 (14.75)	3/10 (30)
S.R^a^	11/41 (26.8)	4/5 (80)	4/9 (44.4)	2/3 (66.7)
Glu^b^	30/41 (73.2)	1/5 (20)	5/9 (55.6)	1/3 (33.3)

^a^SR: spontaneous remission. ^b^Glu: meglumine antimoniate or Glucantime. ^c^VT: *Leishmania* amastigotes (La, Lv, Lb, and Lch) after TLCK treatment and NP-40 extraction.

**Table 7:** Amastigotes antigens in immunoblottings, revealed by 80–100% sera, from patients with active CL, after clinical remission, and healthy controls.

Antigens (kDa) revealed by sera from patients with CL and controls (patients)^a^											
Active (10)				Remission (10)				Control IDR− (7)			
La	Lb	Lv	Lch	La	Lb	Lv	Lch	La	Lb	Lv	Lch
22***	17*	22***	35***	22***	22***	33*	33*	17	17*	29	33*
33*	22***	33*	40***	33*	35***	35***	50*	33*	29*	33*	45
35***	29*	35***	50*	35***	40***	50*	65*	45	33*	45	50*
40***	33*	40***	57*	40***	50*	74*	74*	50*	45	50*	57*
50*	35***	50*	74*	50*	65*	80**	86**	57*	50*	65*	65*
57*	40***	65*	80***	57*	74***	90*	90***	65*	65*	74*	74*
65*	50*	74*	86*	65*	98***	98*	98*	86	90*	86	86*
74***	57***	80***	90***	74***	104***		104***	90	96	90*	98*
80***	65*		98*	100**	108**			98*	128	98*	
98*	74***		104***					128*	132		
100***	80***		128**								
104***	86***										
128*	90*										
132**	98***										
	100***										
	104***										
	115**										

Total number of antigens in immunoblottings											
La	Lb	Lv	Lch	La	Lb	Lv	Lch	La	Lb	Lv	Lch
Active lesions				Clinical remission				Healthy controls			

14	17	8	11	9	9	7	8	10	10	9	8

^a^Common bands present in each *Leishmania* species in active, or remission and control IDR− groups^(∗)^. MW bands revealed by sera from active and clinical remission in same patients, not found by sera from controls at the respective homologous *Leishmania* specie^(∗∗∗)^. Specific antigens found in one leishmania specie only in active or remission group, respectively^(∗∗)^.

**Table 8:** Antigens decreasing in band frequencies revealed by sera after spontaneous remission, Glucantime, or VT treatment in immunoblottings.

Molecular weight (kDa)		
Post-SR	Post-Glu	Post-VT
142	145	142
132	132	120
128	128	96
120	104	80
115	100	57
104	70	35
100	53	29
98	33	22
90	31	20
86	24	17
80	17	15
53	15	13
45		
27		
